# Relative Value of Point-of-Care Ultrasound in Person Specification of UK Consultant Job Advertisements Across Multiple Acute Specialties

**DOI:** 10.7759/cureus.64862

**Published:** 2024-07-18

**Authors:** Muhammad Nabeel Akhtar

**Affiliations:** 1 Department of Acute Medicine, Stoke Mandeville Hospital, Aylesbury, GBR

**Keywords:** uk consultant jobs, point of care ultrasound (pocus), nhs jobs website, acute medicine, internal medicine, emergency medical service, nhs consultant, intensive care specialist

## Abstract

Background

Point-of-care ultrasound (POCUS) skills are very useful in the management of acute patients. It is unknown how highly valued they are at the consultant level. The objective of this study was to investigate the prevalence of POCUS skills being listed as essential or desirable in consultant job advertisements for acute medicine (AM), intensive care medicine (ICM), and emergency medicine (EM) in the United Kingdom (UK).

Methods

We investigated the frequency with which POCUS skills are listed in person specification of consultant jobs advertised in the UK in three acute specialties (AM, ICM, and EM). Data were collected prospectively between May and June 2022 from the NHS Jobs website.

Results

A total of 286 jobs were identified, and 11 jobs (3.85%) listed POCUS skills as either essential or desirable. In AM consultant jobs, only two (1.83%) categorized POCUS as desirable or essential compared to five (11.6%) in ICM and four (3%) in EM.

Conclusion

POCUS skills are mentioned in the person specification in a minority of consultant job advertisements in acute specialties and currently do not seem to be widely viewed as essential or desirable for consultant practice in these specialties. It may be due to not many consultants are currently trained for this new skill, which in turn has led to the scarcity of the skill set demanded by the employing trusts in the UK.

## Introduction

Point-of-care ultrasound (POCUS) is a bedside ultrasound assessment of patients. It is a skill that is becoming increasingly valued as a clinical tool, particularly in acute specialties. It can provide rapid evaluation of respiratory, cardiovascular, and abdominal pathology [1]. With this timely information, doctors can produce more accurate diagnoses rather than relying only on clinical examinations and basic X-rays [2]. There is also less need to wait for formal imaging to initiate appropriate management [3].

In addition to that, POCUS skills have been mandatory for trainees in emergency medicine for some time [4]. They have recently become a mandatory requirement for trainees in acute medicine as well, but it will take years before this cohort finishes training. They are not mandatory for intensive care medicine (ICM) trainees. It is unknown how highly valued these skills are at the consultant level.

## Materials and methods

To conduct this observational study, we manually searched for the person specification for the United Kingdom (UK) consultant job advertisements on the NHS Jobs website. We sorted our scheme of work into categorical steps that filtered out our information according to our requirements.

Amongst the first steps was the process of collection of data, which encompassed everything from extracting data and making it easily accessible. We started out by identifying acute specialties that we wanted to include in our study regarding UK consultant jobs. It was identified that acute medicine (AM), ICM, and emergency medicine (EM) are the most essential ones for our study. In addition to the classification of specialties, we collected only consultant job advertisements from the NHS Jobs website that were posted in May and June 2022. Our selection criteria majorly relied upon the fact that the advertisement must include job specifications and that they must have defined selection criteria for the consultant’s post. We then proceeded to extract the data from those advertisements, and this aided us in finding the value of POCUS skills and in concluding whether or not this is a skill that is essential in consultant jobs.

Furthermore, the second step we took was the identification and definition of variables that corresponded to our area of study. We defined POCUS inclusion by filtering explicit mentions of POCUS skills and ultrasound training requirements [5,6]. The third step incorporated the analysis of the collected data where we comparatively and quantitatively analysed the data. However, throughout the process of data collection and analysis, some factors demanded extreme care and caution. Some limitations made this study relatively challenging. We had to take into account the evolving healthcare trends, regional biases, and variations in job advertisements. Ultimately, after the meticulous steps of data collection, we proceeded to the interpretation of our findings.

The resultant data were analyzed in Microsoft Excel (Microsoft® Corp., Redmond, WA), and data for each specialty were compared using a two-tailed Fisher’s exact test.

No ethical approval was required as this study module focuses purely on the researcher's observation rather than the trial and testing of human or animal subjects.

## Results

**Figure 1:**
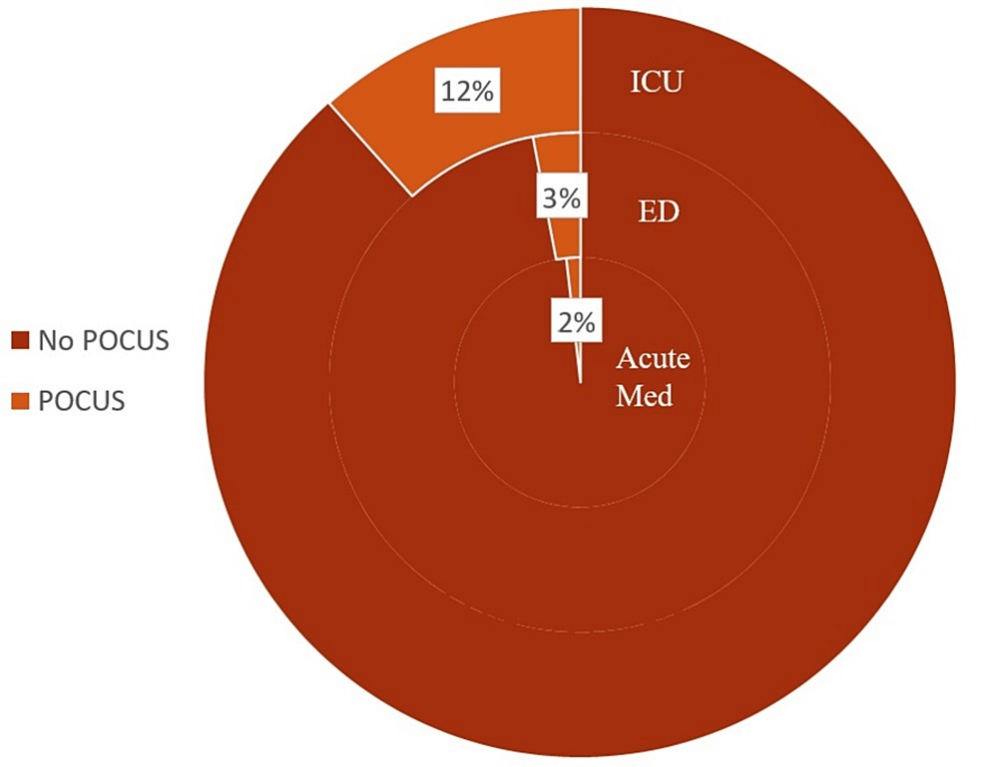
Pie chart showing the percentage of UK consultant jobs with POCUS included in the person specification ED: Emergency department, ICU: Intensive care unit; POCUS: Point-of-care ultrasound

A total of 286 jobs in the relevant specialties were identified in the period (109 AM, 134 EM, and 43 ICM). This revealed that, out of the 286 jobs, only 11 (4%) specifically mentioned POCUS within the person specification. Figure 1 demonstrates the proportions of job advertisements mentioning POCUS across the three specialties. The majority of the jobs do not require POCUS as an essential or desirable skill. To elaborate, out of the 109 AM job advertisements, only 2% (two jobs) mentioned POCUS skills, out of 134 EM jobs, only 3% (four jobs) require it, and out of 43 intensive care jobs, only 12% (five jobs) mention it. Therefore, these statistics justify the fact that most job advertisements do not require POCUS skills.

**Figure 2:**
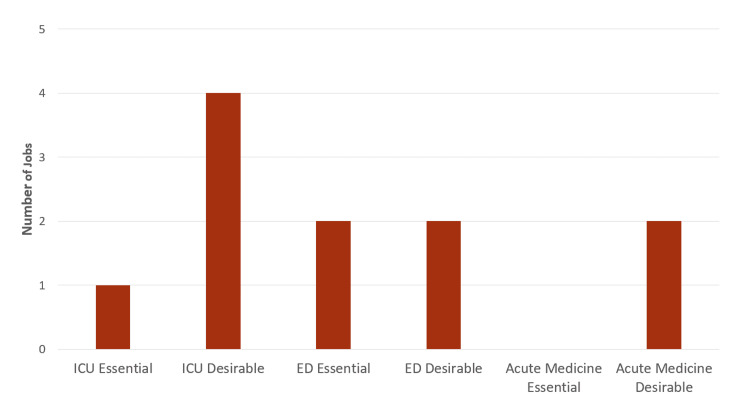
Bar chart with quantitative UK consultant jobs with POCUS in person specification in multiple specialties ED: Emergency department, ICU: Intensive care unit; POCUS: Point-of-care ultrasound

Since we had a relatively small sample size, we used Fisher’s exact test to determine if there was any nonrandom association between the three categories. There was no statistically significant difference between the overall numbers of jobs mentioning POCUS between any of the specialties (Fisher’s exact test: p>0.05) [7]. Figure 2 shows whether POCUS was mentioned in essential or desirable criteria in each specialty. There were no cases in which it was mentioned in both. Since the number of jobs mentioning POCUS skills as desirable or essential ranged from two to five jobs across the three categories, there was no significant difference between the jobs and their categories. 

There was a small but significant difference between the number of jobs listing POCUS skills as desirable between ICM and ED two-tailed Fisher's exact test (p=0.03), but no other differences between these subgroups.

Additionally, Figure 2 elaborates further on the context of POCUS in person specification. This figure highlights and differentiates between POCUS being desirable or essential, out of the 11 job advertisements that mention it in person specification. Out of the five intensive care job advertisements, one mentions POCUS as essential, while four mention it as desirable. Out of the four EM job advertisements, two mention POCUS as essential, two mention it as desirable, and both of the two AM job advertisements mention it as desirable.

## Discussion

This study identified two major insights on POCUS skills. Firstly, POCUS skills are not considered an essential skill for consultant jobs. Secondly, POCUS skills are more relevant to some fields of medicine than others when referring to consultant jobs in the UK. A conclusion that we drew from these results is that POCUS skills pertaining to consultant job requirements are a very subjective matter that highly relies on the institution that is employing the consultant [8]. Some may view it as desirable or necessary, while others regard it as just an additional perk. The one major barrier could be that it is still a new skill and many consultants at the current time are not trained as training for POCUS is a lengthy process but maybe in the next few years. When there is more training available, this skill will become more desirable or essential for consultant job advertisements.

As evident in the figures, POCUS skills are mentioned in person specification in only a minority of consultant job advertisements. Additionally, 11/286 job advertisements mentioning said skills thoroughly emphasize the position of POCUS in the medicine domain. The fact that the world is progressing with its research on POCUS skills has just started to emerge in consultant jobs; however, the progression seems slow [9]. An obvious reason for this could be the absence of a systematic POCUS curriculum or the lack of certified training workshops. The lack of the aforementioned could possibly affect the perception of POCUS skills amongst the medical community as having mediocre skills is beyond hazardous than not having a specific skill at all. Another possible reason could be that most medical practitioners, despite believing that POCUS skills are essential, do not consider it as a distinct discriminator in consultant applications. The overall paucity of consultant jobs mentioning POCUS in the UK across acute specialties may be because POCUS skills are not considered a significant discriminator in consultant applications. The purpose of person specification is to aid in shortlisting a candidate. POCUS skills may be considered less valuable than other forms of clinical experience and thus less amenable to generating a useful shortlisting score. Alternatively, POCUS may be a more nuanced subject that is better considered at the interview rather than within a constrained set of person specification criteria.

The overall paucity of consultant jobs mentioning POCUS in the UK across acute specialties may be because POCUS skills are not considered a significant discriminator in consultant applications.

Furthermore, the three targeted areas of medicine we considered (i.e. intensive care, AM, and EM) are highly dependent upon the traditional methods of diagnosis, such as physical examination, patient history, and laboratory tests [10,11]. The shift from traditional methods of diagnosis to POCUS is a very massive one where not only does the medical practitioner need to learn the skill but also practice it [12]. While most doctors do feel that they are more confident about their diagnosis when they use POCUS, it is still a relatively new and emerging technology that will require some time to get used to. At the end of the day, most consultant jobs require one to be very thorough with fairly common methods (i.e. physical examination, laboratory tests, and other conventional methods) [13]. We deduced the opinion that most of the employing institutions do not value POCUS as a skill that is very essential when it comes to employing a consultant.

Some jobs mentioning POCUS may have had an advertisement created with a particular candidate who has POCUS skills in mind. They may perhaps envisage becoming the POCUS lead of a department and have already highlighted this to the clinical lead in question. This may contribute to the low rate of mentioning POCUS in job advertisements if few candidates are using POCUS as a selling point. Moreover, our data showed that 11 of the 286 jobs that called for POCUS skills as a requirement were very specific job advertisements, while most of the other jobs were very general. To elaborate, some jobs target a wider pool of doctors, while others target a specific pool, and since most of the jobs address general requirements, POCUS is likely considered a very specific skill in their opinion.

Some of the limitations of this study include the evolving healthcare trends, regional biases, and variations in job advertisements. This study was conducted on a small scale, and the period was only 30 days. This does not give fair or concrete substance regarding whether or not POCUS skills are required for consultant jobs. It could be possible that, during the time we chose for this study, only specific consultant jobs were vacant, and they might not necessarily require POCUS. This may not have been a representative sample overall of jobs advertised in these acute specialties. The occurrence of mentions of POCUS in job advertisements may have significantly changed since we undertook this study. Temporal trends would be interesting to investigate, and we would expect the proportion would increase over time as POCUS skills become more common and become a more useful discriminator amongst candidates. However, the rollout of POCUS training in the UK has been slow, largely owing to a relative dearth of POCUS trainers, and it may take some time for such trends to manifest.

Essentially, the question is, are POCUS skills even necessary for consultant jobs? The fact that our study clearly displays that POCUS skills are not a very prevalent requirement for consultant jobs justifies the statement that POCUS skills are, at the moment, not considered essential. Prior to the study, our hypothetical analysis was that consultant jobs would mark POCUS as an essential or desirable skill. However, after surveying and analyzing our results, we concluded that there is an extensive path ahead of POCUS for it to be considered an important skill for consultants and medical professionals [14]. Some ways in which POCUS skills can potentially be made more conventional are i) standardizing a curriculum for POCUS skills, ii) providing access to related equipment, iii) regulation of protocols involving POCUS skills, and iv) cultural and institutional acceptance of this skill. An increase in the number of candidates with these skills may influence their perceived desirability and may increase the proportion of job advertisements that mention POCUS in the future [15]. Once this has been established, this productive skill can aid the hospital environment to cater to the growing patient needs [16].

## Conclusions

To conclude, POCUS skills are very crucial in rapid evaluation and quick diagnosis in emergencies, but these are still not being widely considered as an essential or desirable requirement for consultant jobs in the UK. It may change in the near future considering its underlying importance in rapid diagnosis and treatment in acutely unwell patients. In addition, it is evident that all acute specialties are already making POCUS skills a part of the training curriculum in the UK which, in return, will improve POCUS skills and the quality of the care being delivered to patients.

The relative absence of POCUS skills in consultant job advertisements highlights a disconnect between the current capabilities and advancements in medical practice and traditional frameworks used in hiring. Addressing this gap requires concerted efforts in education, certification, and awareness, as well as a proactive approach to updating job criteria to align with contemporary clinical practices.
